# Diary of Dhaka Dengue Days

**DOI:** 10.4269/ajtmh.23-0822

**Published:** 2024-10-01

**Authors:** Labannya Das Puja

**Affiliations:** Sir Salimullah Medical College and Hospital, Dhaka, Bangladesh

I clearly remember a young woman who came in with her sister-in-law while I, an internal medicine trainee, was on admission duty in the ward. The patient said she had contracted dengue 9 days ago when in Dhaka, which happened to be one of the worst-hit areas of one of the deadliest outbreaks of dengue that our country, Bangladesh, had ever seen. Now, she planned on returning home as she thought she was OK. She had no more symptoms, although she was still feeling tired. She wanted to check if it was OK to stop taking the oral drugs she was given for dengue, or if she needed to take something different for the tiredness. She was a charming, happy lady, laughing and joking with me.

While checking her vitals, I found a very weak pulse. Her blood pressure was non-recordable via my digital blood pressure machine and below 65/45 via my manual blood pressure machine. I thought of the possibility of machine malfunction, as the patient was not exhibiting any extreme symptoms other than day-long tiredness. I requested another available in-house blood pressure machine while counseling the patient and her attendant that it could possibly be shock. As I said this, the patient added that she was pregnant, and she thought the baby was moving much less than usual.

This lovely lady was just one of the many patients I admitted with shock when the fever was no more, and they thought they were entirely OK. From my experience, most patients went through shock during the second week of dengue, after the febrile phase, owing to severe plasma leakage. Hence, platelets and hematocrit were checked carefully. Most of my other patients were complaining of abdominal discomfort, and they frequently had elevated aspartate aminotransferase and alanine transaminase levels, suggesting liver damage. When encountering a patient with dengue, we usually follow the latest national revised pocket guideline after the patient has been thoroughly checked in. Pregnant patients, as well as patients with bleeding, shock, other comorbidities, coinfections, or organ failure or immunocompromised patients should be prioritized. Had she not been prioritized when she presented 9 days ago? Had someone not determined if this pregnant woman had regular dengue fever, the more severe dengue hemorrhagic fever, dengue shock syndromes, or even expanded dengue syndrome? I know everyone in our health sector is trying their best, but did someone miss one of these dangerous things in this woman?

In any event, this was not the time to criticize. I needed to make sure this patient was safe. This laughing, pleasant, pregnant woman would only be thoroughly checked in after she went to the cash receiving area, buildings away, and then had her unit determined for admission. Thinking of the blood pressure needed to perfuse her brain, her smile, and her fetus and seeing my admitting colleague managing a potentially fatal case of poisoning, I decided it didn’t matter who was under whom as long as the patient’s permission, our discussion, and counseling were documented. With that, I focused on saving her life.

I promptly sent her sister-in-law to do the admission procedure. Meanwhile, I quickly documented the patient’s condition, our discussion, her understanding, and the necessary permissions. Moving swiftly, I started shock management via inserting an intravenous channel and starting the first fluid bolus. Sitting at her side and monitoring her health, I phoned the admission processing administration and my colleague, informing them of what I’d done and how I’d gone outside the standard.

While I watched to see if she improved, I couldn’t help thinking that the walls of my hospital have witnessed lots and lots of recorded-unrecorded, endemic-pandemic-epidemic diseases or disasters since its birth in 1875. This tertiary referral hospital is the oldest medical institution in Bangladesh, situated on the Buriganga riverbank. As a government hospital, its services are significantly cheaper than those of private hospitals. More than half of the dengue cases in the country have been reported from Dhaka. All eight units in the two buildings that compose the Medicine Department brimmed to overflowing with patients sick with dengue. All the nonpaying beds, cabins, and paying beds were full. Initially, patients were given white mosquito nets at admission. But the demand had increased more than the supply. Nowadays, it is impossible to distribute mosquito nets to all patients. Although the beds may have had white nets draped over them, there were patients without nets covering the floors and verandas. Patients were even willing to stay right in front of the washrooms and lifts. People of all classes were admitted side by side. Some days, it was challenging for me to find a place to just sit by a patient to measure their blood pressure.

A week or so after I cared for the pregnant woman, I got a text from my Baba (father) during rounds saying, “Your Maa (mother) has a 102° fever.” In spite of being in the midst of a dengue outbreak, I still had hope that it was just a fever, as Maa had been working by the furnace and sweating a lot in this weather. I contacted the diagnostic center to avail the blood collection service from home. I thought my mother would not be able to go out and give a blood sample to the diagnostic center, and I knew of a service where medical technicians go to people’s homes and take blood directly from the patient. I called them to collect blood from my Maa as I kept working. I also told my parents to drink oral rehydration salts and paracetamol urgently. One day later, my Baba developed purpura. We didn’t even know he had a fever. He had weakness 5 days before, but somehow we had not connected things until the purpura appeared. Long story short, their platelet counts dropped. Yes, both of them now had dengue! I knew my hospital had no empty beds. After discussing the situation with the head of my unit, we decided to continue my parents’ treatment at home with strict hydration therapy and medication via phone, according to the guidelines. I kept monitoring their blood pressure when I was at home with them. I taught them how to use a digital blood pressure machine so they could keep track of their blood pressure by themselves when I was on shift. While I was treating dengue patients in the hospital, I kept praying to God, “Please let my parents’ blood pressure be stable!”

Throughout this intense outbreak, we saw people from Dhaka and nearby areas. They came to us via river routes, boats, launches, or bridges. The sheer number of patients compared with the relatively few caregivers made each day challenging. We had honed our guidelines, getting the results of complete blood count, dengue NS1, dengue IgG, and dengue IgM within hours while simultaneously giving bolus and maintenance fluids as dictated by blood pressure. Our consultants discussed when precisely to give blood, when to give colloid fluid, and when to give crystalloid fluid. We advised the lucky ones without warning signs to go home.

Returning to work at the hospital filled with patients with dengue, without mosquito nets, right beside the river, with so much stagnant dirty water lodged all over the area—the probability of becoming a dengue patient of my own worried me. I laughed to myself that with the scarcity of doctors, my team could not afford to lose an active clinician right now. If I got sick, I would probably lie beside another patient in my unit and, with my clinical eye, keep checking to see if anyone was getting lethargic or developing bleeding. That’s how I might still help my teammates. But the burning question would be “Will there be a bed for me?”

It was a miracle that I did not get sick then. I sent a special thanks to my immune system. With time, things were starting to get sorted. Fortunately, my parents got well with no complications. The lady with whom we started our diary and her husband later came to thank us; both the mother and the baby were healthy. In our doctor’s room, we provided conservative treatment to one of our fifth-year students for dengue just before his last of four professional exams. This exam included surgery, obstetrics and gynecology, and medicine. He passed with distinction, and we claimed we contributed a fair share to his success! Jokes apart, he was obviously one of the meritorious students in his batch, and we were really anxious because we did not want him to miss his final examination. All told, between January 1 and November 9, 2023, authorities recorded 1,449 dengue deaths and a total of 287,239 patients. Each of these 287,239 patients had a story, and for everyone who lived, there were people like me who worried about them throughout their whole illness. And for those who died, there were people like me who loved and cared for them.

I would love to say that I made it through this dengue outbreak without losing someone I cared for, but that would not be the truth. I cannot forget the face of one of our beloved medical juniors whom we lost from expanded dengue syndrome. She didn’t want to die. She didn’t have any comorbidities that we knew of. Even now, I keep thinking, “Why her?” It could be me. It could be anyone.

